# Study on Active Particles in Air Plasma and Their Effect on α-Amylase

**DOI:** 10.3390/foods11182896

**Published:** 2022-09-18

**Authors:** Cunshe Chen, Ruohao Sun, Ping Liu, Jufang Yang, Zhixuan Ouyang, Zhihua Pang

**Affiliations:** Beijing Advanced Innovation Center for Food Nutrition and Human Health, Beijing Technology & Business University (BTBU), Beijing 100048, China

**Keywords:** plasma, α-amylase, active particle composition, enzymolysis activity

## Abstract

As a new technology for food processing, plasma has good prospects for protein modification. This study investigated the effect of plasma on the activity of the α-amylase. The composition of the active particles in air plasma generated by spark discharge was analyzed and determined. Furthermore, the quantitative analysis of the active particles such as H_2_O_2_, O_3_, and -OH was made by the chemical detection method. Powdered α-amylase was treated with plasma in various conditions, in which α-amylase and the variation of α-amylase activity under the action of air plasma were quantitatively analyzed. The results showed that the concentration of active particles in the system was positively correlated with the action time for air plasma. After 5 min of plasma action, the concentration of O_3_ and H_2_O_2_ was large enough for food disinfection, but the concentration of -OH was smaller and its lifetime was extremely short. Moreover, it was determined that the optimum action time for the activation of solid powdered α-amylase by air plasma was 120 s. With higher energy, the air plasma acts directly on solid powdered α-amylase to destroy its spatial structure, resulting in enzyme inactivation, sterilization, and disinfection.

## 1. Introduction

The application of plasma technology is growing as theoretical studies into plasma physics and plasma chemistry become more in-depth. Plasma presents a highly excited unstable state, which contains a large number of high-energy electrons, ions, molecules, neutral atoms, excited state atoms, photons, and radicals [1,2,3]. The plasma transforms the electrical energy from the power source into various forms of energy such as light, sound, electricity, and chemistry. The main transporters of its energy are electrons, whose motion and frequent collisions during the gas discharge make them reach equilibrium in a certain state of energy distribution. In the process of plasma excitation, the effects of ultraviolet light, electric field, and heat are also generated, and all these components can interact with biological macromolecules or cells to induce various biological effects together [4,5,6].

When plasma acts on proteins, the activated particles and active substances generated by the plasma through the electric field can act on the protein, promoting intermolecular cross-linking and depolymerization as well as the formation of new functional groups, resulting in subsequent changes in protein structure, leading to changes in the functional properties [7,8,9]. Plasma can also have an effect on the microstructure and surface hydrophobicity of a protein surface. The plasma produces activated particles that impact the protein surface, causing changes in the appearance of the protein [10]. Some studies suggest that the increased hydrophilicity after low-temperature plasma treatment may be related to the introduction of oxygen-containing groups on the sample surface, which can increase the wettability of the sample surface [11,12]. The plasma treatment process also causes changes in the secondary structure of the protein, resulting in the oxidation of amino acid groups by the reactive particles, which ultimately leads to changes in the emulsification, solubility, foaming, and flavor binding ability of the protein [13,14,15,16]. Most of the enzymes are protein in nature and plasma can be used to improve the endogenous enzyme activity, thus affecting storage time, etc [17].

Alpha-amylase, or α-1,4-glucose-4-glucan hydrolase, is commonly found in animals, plants, and microorganisms and acts randomly on starch and glycogen inside molecules to break their α-1,4 glycosidic bonds and hydrolyze them to produce small molecules such as glucose, maltose [18,19], and oligosaccharides. It has been widely used in food, textile, paper, and feed industries, and has an important role in shortening the production cycle, improving product yield and raw material utilization, and improving the product quality and saving food resources, etc [20]. It has become an essential key enzyme preparation in industrial production. Enzyme activity is crucial in the application of α-amylose.

In order to investigate the effect of plasma on the enzymatic activity of powdered α-amylase under different conditions, spark discharge was used to generate plasma. Furthermore, the produced active particles were analyzed and their composition was determined. The H_2_O_2_, O_3_, -OH, etc. were quantified using a chemical detection method. The purpose was to better understand the composition of the active particles and explore the conditions that maximize the activity of α-amylase to discover ways to quickly boost the enzyme activity. α-amylase was used as a bioindicator to study the interaction between air isotopes and biological macromolecules in order to direct the application of air plasma to cold chain food disinfection.

## 2. Materials and Methods

### 2.1. Experimental Materials

Crystalline iodine, potassium iodide, citric acid monohydrate, disodium hydrogen phosphate dodecahydrate, potassium bromide, sodium thiosulfate, concentrated sulfuric acid, soluble starch, potassium dihydrogen sulfate, disodium hydrogen phosphate, borax, and sodium hydroxide, were all obtained analytically pure from the Beijing Chemical Factory; soluble starch was obtained analytically pure from the Beijing Aoboxing Biotechnology Co., Ltd. (Beijing, China); α-amylase was obtained from the Beijing Aoboxing Biotechnology Co., Ltd. (Beijing, China); sodium indigo disulfonate was obtained form the Sinopharm Chemical Reagent Company BS (Beijing, China); sodium calcium reagent carboxylate was obtained from the Sinopharm Chemical Reagent Company Ind (Beijing, China); and malachite green was obtained from the Beijing Chemical Reagent Company AR (Beijing, China).

### 2.2. Equipment and Instruments

A plasma generator type A (Sanfish Electric Equipment Co., Ltd., Hangzhou, China), UV-visible spectrophotometer UV2550 (Shimadzu Japan, Chengdu, China), digital display thermostat water bath HH-2 (Jiangsu Jereel Electric Co., Changzhou, China), high-speed frozen centrifuge Himac CR 22G (Hitachi, Tokyo, Japan), an electronic balance Model JA5003 (Shanghai Precision Scientific Instruments Co., Shanghai, China), test tubes, triangular flasks, beakers, volumetric flasks, centrifuge tubes, glass rods, timers, cuvettes, and four layers of gauze were all of the instruments used in the experiment.

### 2.3. Experimental Methods

Plasma in the excitation process forms chemically active electrons, ions, and radicals, as well as active atoms, excited atoms, excited molecules, and active molecular fragments, etc. These active particles have the characteristics of a good chemical activity, short life cycle, and when particles are in contact with other substances they are easy to react with them. So the number of active particles generated by the plasma can only be reflected by indirectly measuring the number of its reaction products. Therefore, the indirect chemical detection method was the preferred method for the experiments.

#### 2.3.1. Quantitative Analysis of Ozone Production from Air Plasma

The ozone detection was conducted according to H. Bader’s method [21], with some modifications. The absorbance values of the solutions were determined by a UV-Vis spectrophotometer at 610 nm using water as a reference. The plasma was generated by excitation at 2 cm from the liquid surface, and the time of the plasma formation was controlled as 0, 1, 2, 3, 4, 5, and 8 min. The solution containing sodium indigo disulphonate was used as the absorbent solution, and the absorbance value of the absorbent solution was measured at 610 nm after the plasma action.

#### 2.3.2. Quantitative Analysis of Hydrogen Peroxide Production by air Plasma

The quantification of plasma-generated hydrogen peroxide was done using Zhu’s method [22], with some modifications. The solution of calcium reagent sodium carboxylate (concentration) and boric acid–sodium hydroxide buffer solution (concentration) was mixed by the ratio of (volume proportional gradient). The absorbance values of the solutions were measured by a UV-Vis spectrophotometer at 630 nm and a standard curve was made.

The sample of 5 mL of the calcium reagent sodium carboxylate absorbent solution (concentration) was poured into a test tube and excited the plasma at 2 cm from the liquid surface. The time of the plasma formation was controlled as 0, 1, 2, 3, 4, 5, and 8 min, respectively. The absorbance values were measured at 630 nm. Three parallel actions were taken at each time point and the average value was obtained. The hydrogen peroxide production was calculated based on the standard curve.

#### 2.3.3. Quantitative Analysis of Hydroxyl Radical Generation from Air Plasma

The quantification of plasma-generated hydroxyl radicals was carried out using E. Peralta’s method, with some modifications [23]. Disodium hydrogen phosphate–citrate buffer (pH 4.0) and malachite green solution (1.0 × 10^−4^ mol/L) were prepared. An aliquot of 0.5, 1.0, 1.5, 2.0, 2.5, 3.0, 3.5, and 4.0 mL of malachite green solution was mixed with the phosphate buffer. The absorbance value was measured at 620 nm, and the standard curve was plotted against the concentration of malachite green.

For the quantification of the hydroxyl radical generation, 0.2 mL of the malachite green solution, 2.4 mL of the phosphate buffer solution (PBS), and 1.0 mL of ultrapure water were filled in each glass tube. The glass tubes were held in place using a test tube holder and the plasma treatment was carried out as above. The absorbance values were measured at 620 nm with a UV-Vis spectrophotometer immediately after the auction and the hydroxyl radical generation was quantified from the standard curve.

#### 2.3.4. Determination of α-Amylase Enzyme Activity

The α-amylase enzyme solution was prepared and the enzyme activity of α-amylase was determined under different action conditions and times. The absorbance value was measured at 660 nm using a UV-Vis(Ultraviolet–visible spectroscopy) spectrophotometer. From the standard curve, the residual starch concentration x was calculated, and the enzyme activity of α-amylase was obtained by the following methods.

Preparation of solutions:

Iodine stock solution: 1.1 g of potassium iodide was dissolved in a small amount of water. A weight of 0.55 g of crystalline iodine was added to the solution and stirred until the crystalline iodine completely dissolved. The resultant solution was diluted with water, we fixed the volume to 25 mL, and stored it in a brown bottle away from light.

Iodine dilution solution: 4.0 g of potassium iodide was dissolved in water and we added 0.4 mL of iodine stock solution, diluted it with water, and made it up to 100 mL.

Phosphate buffer solution (pH = 6.0): 45.23 g of disodium hydrogen phosphate dodecahydrate and 8.07 g of citric acid monohydrate was dissolved in water, and the volume was fixed to 1000 mL.

Soluble starch solution (1 g/L): 0.1 g of soluble starch was poured into 90 mL of boiling distilled water, continuously heated until it completely dissolved, we cooled it, and then made the volume up to 100 mL using 0.1 mol/L of dilute hydrochloric acid.

Plotting standard curves:

Eight test tubes were labeled and the reagents and corresponding doses were added in turn as shown in Table 1.

The solution in test tube 0 was used as a standard blank, and the absorbance values of the solutions in each test tube were measured at 660 nm using a UV-Vis spectrophotometer.

According to Lambert’s law, the absorbance value is proportional to the concentration of the sample in a certain concentration range. It can be seen that the absorbance values of starch concentrations in the range of 0.1–0.8 mg/mL at 660 nm were linearly correlated with the concentration of the standard solution. The linear regression equation for the starch standard solution was obtained from the standard curve as y = 0.0801x−0.0835 with a correlation coefficient of R^2^ = 0.9999.

From the standard curve, the absorbance value of each solution corresponds to the concentration of starch in that solution (the amount of starch left over from the reaction). The amount of starch consumed by the α-amylase reaction was derived from this starch concentration, and the enzyme activity of α-amylase can be found by substituting the formula.

Calculation of α-amylase enzyme activity:

Medium temperature α-amylase enzyme activity unit definition: the amount of α-amylase that liquefies 1 g of soluble starch for 1 h at 60 °C and pH = 6.0 is 1 enzyme activity unit, expressed as U.

Enzyme activity = enzyme activity of 0.1 g/L enzyme dilution solution × dilution times, i.e.,

Enzyme activity = (4 − 7.2x) × N × M × 10^4^ × 10^−3^ (1)

x: the remaining starch concentration in the reaction system (mg/mL), which was derived by putting the reaction solution OD660 nm in the standard curve. The volume of the reaction liquid is 7.2 (mL). Four is the total starch content (1 g/L × 4 mL in mg).

N: the volume ratio of 1 mL to 0.2 mL of enzyme solution is 5.

M: the ratio of 1 h to the reaction time t (5 min) in the definition is 12.

10^4^: the dilution of multiple of the reaction solutions.

10^−3^: unit conversion.

#### 2.3.5. Action of Air Plasma on α-Amylase

A sample of 0.100 g of α-amylase was weighed in an amperometric tube and a spark discharge was used to generate air plasma to act on the α-amylase with action times set to 0 s, 30 s, 60 s, 90 s, 120 s, 150 s, 180 s, and 300 s, respectively. Three parallel experiments were performed at each time point. The same 10 s interval discharge method was used to reduce the effect of the excessive heat generated by the thermal effect of the plasma on the α-amylase. After the treatment, 0.0100 g of α-amylase was taken and dissolved by heating it in a phosphate buffer water bath (60 °C), filtered to remove any insoluble impurities in the α-amylase, and the volume was fixed to 100 mL to obtain the enzyme solution to be tested after the plasma action.

#### 2.3.6. Effect of Low-Pressure Low-Temperature Plasma on α-Amylase

The α-amylase was put in amperometric tubes and plugged with skimmed cotton at the seal to prevent the amylase powder from being sucked away during vacuuming. The amperometric tubes were connected to the vacuum system and plasma was generated by spark discharge to act on α-amylase for 0 s, 30 s, 60 s, 90 s, 120 s, 150 s, 180 s, and 300 s, respectively. Three parallel experiments were performed at each time point. The 10 s interval discharge method was used to reduce the effect of the excessive heat generated by the thermal effect of the plasma on the α-amylase.

A weight of 0.05 g of α-amylase was taken after the action and dissolved by heating with the phosphate buffer solution in a water bath at 60 °C, followed by filtration. The phosphate buffer was used to prepared 10^−4^ of enzyme solution for the measurement of the enzyme activity.

### 2.4. Data Processing

SPSS Statistics software22.0 (SPSS, Chicago, IL, USA) was used to analyze the difference and level of significance of the experimental data. Origin 8.5.1 (Origin Lab, Northampton, MA, USA), and Excel software2019 (Microsoft, Redmond, WA, USA) was used to make the graphs.

## 3. Results and Analysis

### 3.1. Quantitative Analysis of Plasma-Generated Ozone

**Figure 1:**
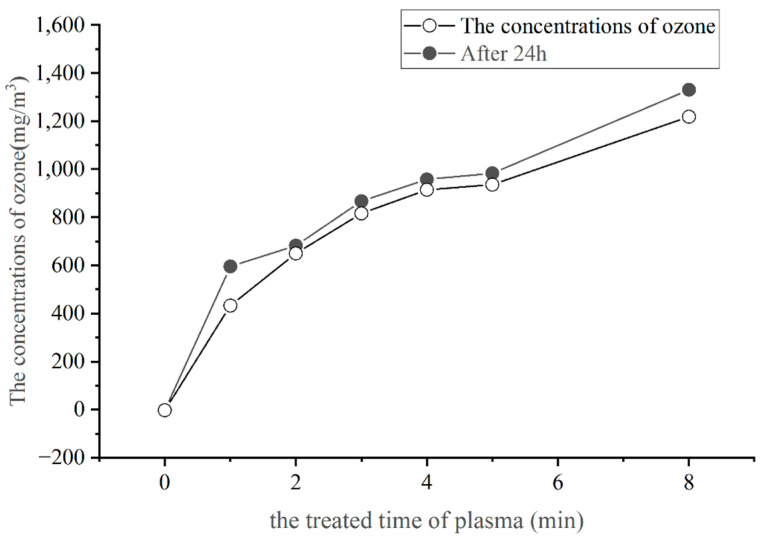
The quality of ozone produced by the plasma.

The standard curve for the ozone was determined using the sodium indigo disulphonate spectrophotometric method, and the standard curve yielded R^2^ = 0.9999 (data not shown). The concentration of the ozone produced by the plasma is shown in Figure 1.

As shown in Figure 1, the amount of ozone produced increases as the plasma action time increases. The ozone produced by the plasma was 0.99, 1.44, 1.82, 2.05, and 2.11 times the ozone content of the air in 1 to 5 min, and 2.73 times the ozone content of the air in 8 min. After the treated solution had been left at room temperature for 24 h, the quality of the ozone absorbed by sodium indigothionate increased, but the trend was essentially the same as at the very beginning. A linear relationship was observed between the magnitude of the ozone concentration generated by the air plasma and the time of action; the longer the enrichment action, the greater the amount of ozone. Furthermore, the combination of ozone and sodium indigothione disulfonate can produce stable compounds. The choice of sodium indigothione disulfonate as the ozone absorbing solution was made because of its specificity, high detection sensitivity, susceptibility to sample interference, and low sample stability. In addition, sodium indigo disulphonate has an adsorption effect on trace amounts of ozone in the air. After the samples had been left in the air for 24 h, the amount of ozone enriched in all application sites increased by approximately the same amount. However, the ozone is unstable and tends to decompose into oxygen at room temperature and when under atmospheric pressure. Therefore, during the plasma generation of the ozone, some of the ozone was decomposed before it was absorbed by sodium indigo sulfonate. So the measured amount of O_3_ was slightly less than the actual amount of O_3_ produced. The quantitative analysis of the ozone is essential for subsequent studies as ozone has a strong oxidizing capacity and its redox potential is second only to that of fluorine. Thus, it can react with most substances in the system and its levels affect the disinfectant and germicidal performance of the air plasma.

### 3.2. Quantification of Hydrogen Peroxide Produced by Plasma

**Figure 2:**
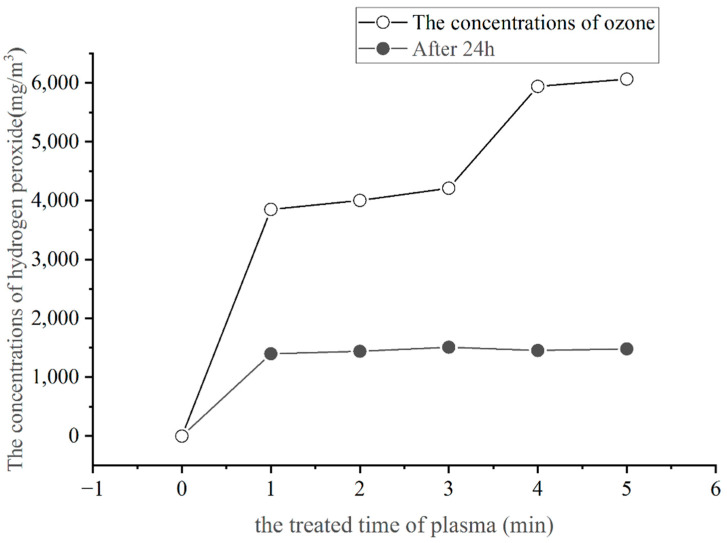
The quality of hydrogen peroxide produced by the plasma.

The amount of hydrogen peroxide was determined spectrophotometrically using the calcium reagent sodium carboxylate, and the standard curve yielded R^2^ = 0.999. The concentration of hydrogen peroxide produced by the plasma is shown in Figure 2.

As shown in Figure 2, the air plasma effect can produce the active material of hydrogen peroxide, and its effect is to show S-shaped growth with the extension of the generation time of the hydrogen peroxide content in the plasma. The amount of H_2_O_2_ generated in the growth of 0–1 min is the largest, then gradually flattened; after minutes there is another growth, and the slow growth tends to be stable after 4 min. The experimental results showed that the amount of hydrogen peroxide generated by the air plasma is influenced by the action time and action environment. The amount of hydrogen peroxide generated by the plasma generally tends to rise with the extension of the action time, but the phase characteristics are obvious. The air plasma has an acidification effect, and the longer the plasma action time, the more obvious the acidification effect. Since the pH of the solution affects the stability of hydrogen peroxide, the stability of hydrogen peroxide is higher in acidic solution, and the alkaline solution is easy to decompose. However, because the calcium reagent sodium carboxylate, as an azo reagent, has the greatest absorption capacity in an alkaline environment, at this time the hydrogen peroxide can be absorbed in the greatest capacity. The increase in the amount of hydrogen peroxide during the 3–4 min’ air plasma action time may be due to the relative equilibrium of the acid–base environment at that time, which is suitable for the oxidation of hydrogen peroxide to break the azo double bond of the sodium–calcium reagent carboxylate. The acidity of the solution and the quantity of hydrogen peroxide increased with the rising plasma action time. The declining pH trend and the amount of hydrogen peroxide both leveled off with further increases in the plasma action time, indicating a correlation between the two. In Figure 2, no significant difference was found in the amount of hydrogen peroxide consumed by the sodium–calcium carboxylate reagent after 1–5 min of plasma treatment and after being left for 24 h. The possible reason could be that the reaction of the sodium–calcium carboxylate reagent with hydrogen peroxide, where hydrogen peroxide oxidation breaks the azo double bond of the sodium–calcium carboxylate reagent, is partially reversible. Hydrogen peroxide is unstable, readily decomposes to form H_2_O, and is not catalytically active.

### 3.3. Quantitative Analysis of Hydroxyl Radicals Produced by Plasma

**Figure 3:**
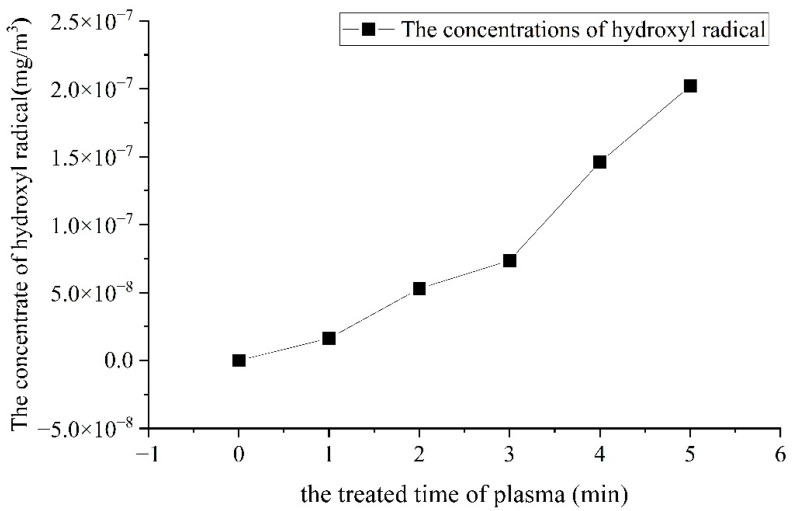
The quality of hydroxyl radical produced by the plasma.

The number of hydroxyl radicals was determined by malachite green spectrophotometry, and the standard curve yielded R^2^ = 0.9993. The amount of hydroxyl radicals produced by the plasma is shown in Figure 3.

As can be seen from Figure 3, plasma can produce hydroxyl radicals, and there was a linear positive correlation between the length of plasma action and the number of hydroxyl radicals produced. The longer the plasma action time, the more hydroxyl radicals were produced. However, malachite green has a strong ability to trap hydroxyl radicals, but they can only be introduced to the plasma system in order to reduce experimental error because hydroxyl radicals have an extremely short lifetime and their generation process is also the start of quenching. In addition, the plasma simultaneously produced active particles such as ozone, hydrogen peroxide, and hydroxyl radicals. In aqueous solutions, the ozone produced by the plasma was unstable and decomposed to produce -OH, -OOH, and O_2_. Therefore, the values of -OH measured in Figure 3 are partly produced by the plasma and partially obtained by the decomposition of ozone. -OH is a free radical with a strong oxidizing power. It is capable of electron transfer, hydroxylation reactions, and the capture of hydrogen atoms from the external environment. Therefore, the quantitative analysis of plasma-generated -OH provides an analytical tool for future studies of the mechanism of plasma action on macromolecular substances and organisms.

### 3.4. Effect of Plasma on the Enzymatic Activity of Powdered α-Amylase

#### 3.4.1. Effect of Air Plasma on the Enzyme Activity of α-Amylase Solid Powder

**Figure 4:**
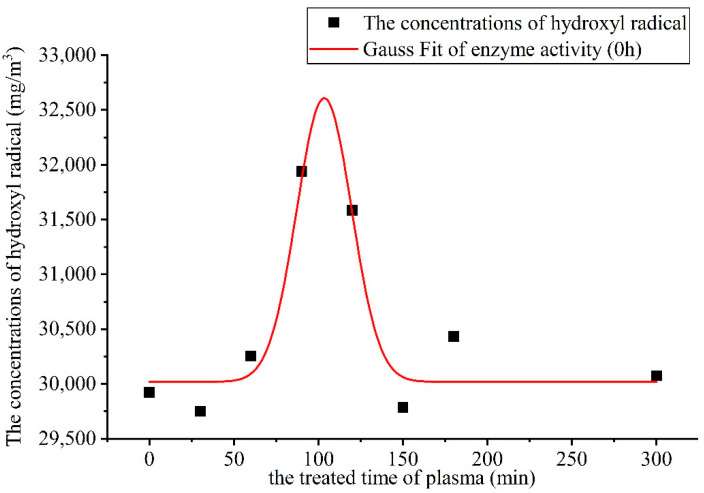
The relationship between α-amylase enzyme activity and duration of action.

**Figure 5:**
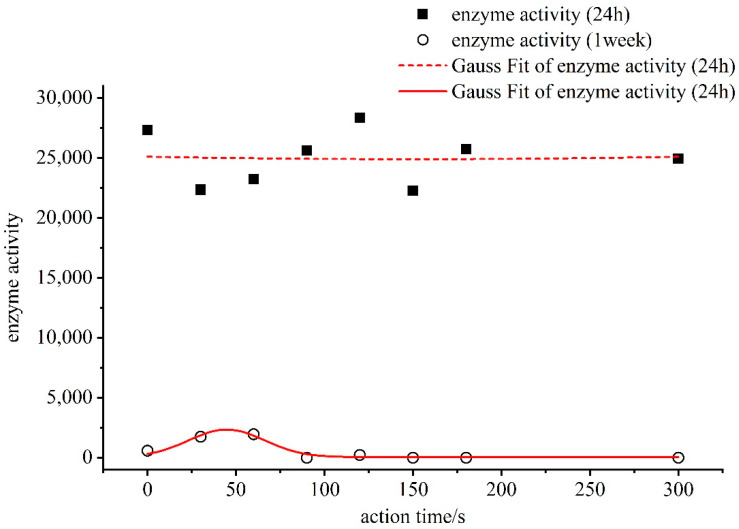
The diagram of α-amylase enzyme activity affected by plasma after 24 h and 1 week.

The air plasma was generated at room temperature and under pressure, and the medium of action was the air. The enzyme activity after the plasma action is shown in Figure 4 and Figure 5.

As can be seen from Figure 4, the change in the activity of α-amylase by the air plasma increased the enzyme activity in the range of 60–120 s. The curve of change in the enzyme activity was fitted from the origin and the greatest increase in enzyme activity was observed at 100 s. The air plasma produced relatively high concentrations of O_3_, hydrogen peroxide, -OH, and other active particles during excitation and formation. Particularly, O_3_ and hydrogen peroxide exhibited strong oxidative activity and can easily interact with each other to influence the reaction on the molecular surface of α-amylase, resulting in a dramatic change in its conformation and activity center. At 120 s of air plasma action, the amylase activity was 31,587 U/mL, which was 5.6% higher than that of the control group.

As can be seen in Figure 5, the enzyme activity of all the samples changed considerably after 24 h of storage following the air plasma action. In the beginning, the enzymes that had been activated were gradually degraded. The enzyme activity was lower than the control group at all points except at 120 s, when the enzyme activity of the samples was 3.75% higher than that of the control group. At 150 s, there was the most severe degradation, with a reduction in 18.49% compared to the non-plasma actuated group. When the samples were left in the greenhouse for another week, it was found that the difference between the 30–120 s was higher than the control group under the action of the plasma, indicating that plasma activates the self-healing system within the enzyme molecule, leading to an increase in enzyme activity. However, when the plasma severely damaged the molecular structure of the α-amylase, this restoration could not take place, resulting in a reduced activity or inactivation of most of the enzymes.

#### 3.4.2. Effect of Low-Pressure Low-Temperature Plasma on the Enzyme Activity of α-Amylase Solid Powder

**Figure 6:**
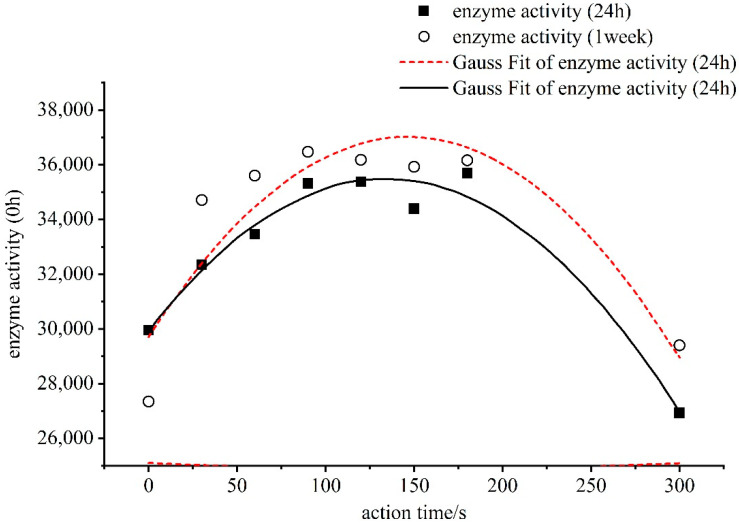
The relationship between α-amylase enzyme activity and duration of action.

The action conditions of the low-pressure low-temperature plasma was 0.1 mbar. The enzyme activity after the plasma action is shown in Figure 6. The proportion of α-amylase activity increased by plasma is shown in Table 2.

The solid dots and lines in Figure 6 show the change in enzyme activity when the plasma action amylase was just used up, whereas the hollow dots and dashed lines show the measurement of α-amylase activity after the plasma action was completed and left for 24 h. As can be seen from Figure 6, the α-amylase activity was strongly influenced by the time of the plasma action, and the pattern of change in the amylase was the same as after 24 h of sitting. The fitted enzyme activity curve showed a parabolic form of increasing and then a decline. At 120 s of plasma action, the enzyme activity reached a reasonable maximum of 35,362 U/mL and 36,225 U/mL, respectively. Firstly, α-amylase is a special protein with highly specific catalytic activity. The catalytic activity and stability of amylase are greatly influenced by environmental factors. In this system, the plasma is formed at low temperatures and under certain vacuum conditions. It is a thermodynamically non-equilibrium plasma with high electronic energy, low ion and gas temperatures, and high active ion energy. When the α-amylase molecule is exposed to the plasma field, it maintains a sufficiently high chemical activity at low temperatures and various active particles interact with it, changing its spatial conformation and activity center. In Figure 6, the enzyme activity starts to decrease after the 180 s of plasma action. At 300 s of plasma action, the α-amylase activity decreased by 10.1% compared to the control. However, after 24 h, the enzyme activity rebounded slightly, but it was not higher than the control. A possible explanation is that the active particle field formed by the plasma caused some damage to the enzyme structure, and if the plasma action continued, the α-amylase would have been completely inactivated.

Secondly, the α-amylase activity at the end of the treatment was compared with the activity for 24 h at room temperature. Although the general trend of change was similar, the phenomenon of stress self-repair and the activation of the enzyme only occurred after 24 h. The plasma-treated α-amylase was higher than the α-amylase after 24 h. The possible explanation is that the enzyme has a self-healing function, as a special enzyme, the active site of the protein is greatly influenced by the external environment. As a result, when the conformation of the enzyme changes, the change in the activity of the enzyme molecule will initiate its internal molecular repair system to reduce the larger change in conformation to maintain its stability. Then, the plasma can activate the active group of the enzyme molecule or change its active center to adapt the change in the external environment. Thus, indicating that the enzyme molecule has a strong enzymatic hydrolytic activity.

**Figure 7:**
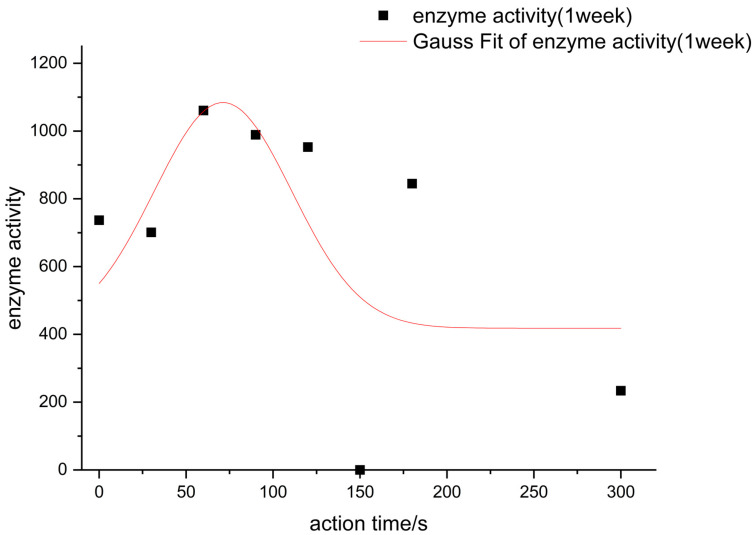
The diagram of low-pressure low-temperature α-amylase enzyme activity affected by plasma after 1 week.

In Figure 7, the catalytic activity of the α-amylase changed considerably when it was placed under the greenhouse for one week after plasma action. The maximum value of enzyme activity at 90 s of plasma action was 988.76 U/mL, which was 34.15% higher than that of the control group under the same conditions. However, after 150 s of plasma action, the enzyme activity decreased in all sample groups and was lower than that of the control group under the same conditions. Therefore, the optimum action time for the α-amylase solid powder was 90 s under low temperature and low-pressure plasma activation conditions.

## 4. Conclusions

The plasma produced in water can be excited to form O_3_, H_2_O_2_, and -OH, as well as oxygen atoms in different excited states, photons, electrons, and various reactive groups. The active particles (O_3_, H_2_O_2_, -OH, etc.) produced by the plasma were unstable and decomposing simultaneously. When the trapping agent is not captured in time, some of the active particles have burst. Where O_3_ could be clearly smelled as irritating odor when the plasma was excited, and the concentration of O_3_ produced was large, -OH had a very short lifetime and small concentration.

The optimum action time for the activation of solid powdered α-amylase using air plasma was 90–120 s. Air plasma acted on solid powdered α-amylase with an action time of more than 150 s. The active particles produced by the air plasma exhibited a larger energy, which had a greater impact on the structure of the α-amylase and can destroy the structure of the enzyme to a greater extent. The treatment time of 90 s under the low-temperature low-pressure plasma treatment can maximize enzyme activity.

## Figures and Tables

**Table 1:** Preparation of standard solutions of starch reagent.

No.	0	1	2	3	4	5	6	7
1 g/L soluble starch solution (mL)	0.0	0.5	1.0	1.5	2.0	2.5	3.0	3.5
Distilled water (mL)	5.0	4.5	4.0	3.5	3.0	2.5	2.0	1.5
Starch standard solution concentration (mg/mL)	0	0.1	0.2	0.3	0.4	0.5	0.6	0.7
Take starch standard solution separately	1 mL							
Add iodine dilution separately	5 mL							

**Table 2:** The proportion of α-amylase enzyme activity increased by plasma.

Plasma action time/s	0	30	60	90	120	150	180	300
Increase in enzyme activity at the end of the action/%	0	8.2	11.8	18.1	18.2	14.9	19.3	−10.1
Increase in enzyme activity after 24 h/%	−8.7	16.1	18.9	22.1	21.1	20.0	20.9	−2.0

## Data Availability

The data presented in this study are available on request from the corresponding author.
